# A sociological investigation of the effect of cell phone use on students' academic, psychological, and socio-psychological performance

**DOI:** 10.3389/fpsyg.2025.1474340

**Published:** 2025-06-19

**Authors:** Xiaoping Tang, Zijun Shen, Muhammad Idrees Khan, Qiong Wang

**Affiliations:** ^1^School of Economics, Guizhou University, Guiyang, Guizhou, China; ^2^Department of Foreign Languages, Sichuan University of Media and Communications, Chengdu, China; ^3^School of Foreign Languages, Guizhou University, Guiyang, Guizhou, China

**Keywords:** mobile phones, students, academic performance, social behavior, mental health

## Abstract

This research investigates the relationship between the use of mobile phones in terms of the academic performance and mental wellbeing of the students who are attending two universities and related high schools in Guizhou province, China. The research aims at gaining insight as to the nature of the relationship between mobile phone use and students' academic, psychological and socio-psychological experiences by using a quantitative cross-sectional survey design. A sample of 300 students (150 students from Guizhou Normal University and 150 from Guizhou University) was chosen for the present study. Information provided by respondents was based on a structured questionnaire scale using five-point Likert scale measuring issues such as mobile phone use habit, academic performance and wellbeing. To enhance the understanding of these associations, moderating variables, such as age, gender, and academic discipline (STEM vs. humanities) were specified for explaining their impact on the use and impacts of mobile phones on students. The Chi-square and Gamma tests were employed in statistical analysis to investigate the interrelationships among the study variables. The finding disclosed that excessive use of mobile phones for entertainment (social media, music, and messaging) is associated with a negative impact on academic grades and mental illnesses (Gamma = −0.09). In contrast, the use of mobile phones among the students for academic purposes such as accessing educational materials had a desirable academic implication (*P* = 0.01). The study underscores the need for mobile phone usage moderation to increase academic achievement and the wellbeing state.

## Introduction

The integration of mobile phones into daily life has significantly impacted students' social interactions, psychological wellbeing, and academic performance. This section explores the social, psychological, and academic implications of mobile phone usage among students. The study also considers the role played by socioeconomic background, access to educational technologies, and different educational levels (e.g., between high school and university learners) in influencing relationships between mobile phone use and student outcomes. The socioeconomic background may condition the way in which mobile phones have an impact on social interactions, as economic status differences generate variations both in terms of students' access to mobile devices, and experiences of connectivity.

### Social implications

#### Social interaction and communication

Mobile phones facilitate constant connectivity, altering the nature of face-to-face interactions among students. While they enable maintaining relationships beyond physical proximity, they may also reduce the quality of in-person communication (Hartley et al., 2023; Campbell and Park, 2008).

#### Social identity and peer influence

Mobile phones play a crucial role in constructing social identity among students through online platforms and social media. Peer influence via mobile-mediated communication can significantly affect attitudes, behaviors, and perceptions within university contexts (Gündüz, 2017; Hartley et al., 2023). The difference in the way that the students engage with social media and the influence from peers can be gender and age specific, and younger students may feel greater peer pressure than the older ones.

### Psychological implications

#### Psychological wellbeing

Excessive mobile phone use is linked to higher levels of stress, anxiety, and sleep disturbances among students. The phenomenon of nomophobia, or the fear of being without a mobile phone, is an emerging concern affecting emotional stability (Thomée et al., 2011; Yildirim and Correia, 2015). Moderators such as gender and difference in academic focus (STEM vs. humanities), may influence how these instances of psychological concern manifest themselves, and students in STEM fields may experience increased pressure to remain connected with their studies.

#### Cognitive effects

Multitasking between mobile phone use and academic tasks can impair cognitive abilities and academic performance. Additionally, dependency on mobile phones for information retrieval may diminish critical thinking skills and hinder information processing (Junco and Cotten, 2012; Kuznekoff and Titsworth, 2013). This association can also be influenced by attributes of age and type of course. By way of example, younger students and those attending classes in humanities may be more mobile phone dependent for non-academic purposes, conversely, students pursuing STEM fields tend to use mobile devices mainly for academic purposes.

### Academic implications

#### Learning and performance

Mobile phones provide opportunities for learning outside traditional settings through educational apps and resources. However, their presence in classrooms can be distracting and disruptive to the learning environment (Chen et al., 2020; Duncan et al., 2012). Students with more access to mobile-based educational tools, typically associated with their socioeconomic status, might benefit much more from such learning possibilities.

#### Academic integrity

The ease of access to information via mobile phones facilitates academic misconduct, such as cheating and plagiarism during exams (Walters and Hunsicker-Walburn, 2015). Research indicates that students who extensively use mobile phones tend to achieve lower grades, report higher anxiety levels, and feel less happy compared to their peers who use mobile phones less frequently. Smith also demonstrated that frequent mobile phone users experience higher levels of anxiety and achieve lower GPAs (Hashemi et al., 2022). Such external factors as course design, students' academic status, and students' exposure to modern educational instruments could operate, with high school students perhaps being subject to exaggerated threats in the field of academic integrity because of their lack of experience in handling academic responsibilities.

### Post-pandemic contextualization

COVID-19 brought some changes in students' relationship with mobile devices especially regarding remote learning, digital tools integration, etc. (Madigan et al., 2022). Since when educational establishments moved to online platforms, mobile phones were very useful in getting learning materials, attending virtual classes and submitting assignments. This switch resulted in significant increases in the screen time posing alarm to the effects of such intervention on students' academic, mental health, and socialization skills (Wong et al., 2021). The rapid implementation of remote learning during the pandemic emphasized at the same time advantages and challenges of mobile device use in education. On the positive side, learning through mobile phones made possible easy access to a vast number of educational resources, and it made tailor-made learning as well as a communication channel between students and educators. But, with the rise in screen time, there were several problems which arose. Studies found that exposure of screens to students has adverse effects on students' mental health with increased cases of anxiety, depression and sleep disturbances (Nakshine et al., 2022). Additionally, the blending of work and life parameters resulted in digital fatigue and burnout on students' side (Neophytou et al., 2021).

Students' academic performance and social interactions were also affected by transition to online education. Although mobile devices offered flexibility in learning they came with distractions which could derail academic focus. Studies show that students who regularly use mobile phones for other purposes apart from learning, during study time, have lower academic and resolution rates (Ibrahim and Jibia, 2024). In addition, the dependency on digital communication platforms limited the face-to-face interactions, and people felt isolated and social skills' development experienced drop (Marinucci et al., 2022).

Still, there are some advantages of mobile devices as well, in the educational context. When applied adequately, smartphones can bring many enriching experiences to learning by offering access to engaging educational apps, creating shared projects, and ensuring independent learning. In addition, mobile technology can help to fill the holes in education by providing students with remote or underserved areas, with resources to make education inclusive (Moe, 2024).

### Review of literature

In the last few years, how smartphones and the internet have affected the social lives of the youth, and their psychological state has become a subject to research. A profound understanding of the impact of digital technology integration on academic, emotional, and social wellbeing has become a critical issue as digital technology integration becomes progressively complex. This review studies in the last few years (2023–2025) are incorporated to analyze the changing settings of smartphones.

Several studies have highlighted the growing global expenditure on mobile phone services, including voice communication, text messaging, and internet access. This trend has prompted questions regarding the sustainability of such usage patterns. One study conducted with a sample of 208 participants adopted a cross-sectional design to examine the influence of socio-psychological factors on sustainable mobile phone usage behavior. Statistical analyses, including *t*-tests, ANOVA, and hierarchical regression, revealed that overall sustainable usage behavior was relatively low among users. Significant differences were observed based on demographic variables such as gender, educational attainment, and residential location. The study also examined the role of personality traits, finding that conscientiousness and neuroticism were particularly relevant in shaping mobile phone usage patterns. However, in composite analysis, demographic factors showed a stronger predictive value than personality dimensions. These findings suggest that promoting sustainable mobile phone use may require interventions targeting demographic-specific behaviors and attitudes, alongside broader behavioral change strategies (Nwanzu and Babalola, 2023).

Further research argues paramount importance of sociopsychological factors in sustainable mobile phone use. Using a mixed-method design, the research found that social factors and knowledge of the sustainability of mobile phones have positive effects on user responsibility. These researchers highlight that age and educational level are the most important elements that determine the way in which students incorporate mobile phones into their academic and social life. Both studies emphasize the importance of considering the various modes in which mobile phones contribute to the development of young people in a school and social setting (Nwanzu and Babalola, 2023).

The focus of research is mostly on the negative implications of excessive smartphone use, but an increasing amount of research start uncovering the positive sides of mobile technologies. For example, Trivedi research explores positive impacts of mobile phones on the learning and achievement of students as a way they improve communication, cooperation and access to educational materials. The deployment of mobile phones eases personalized learning experiences which serve mobile learning and increased information retention among the students. This has become particularly relevant in the post-pandemic world where mobile phones have come to be among the most important tools for remote education and control over academic routines. Depending on whether the students are studying in STEM or humanities, the mobile phone can support learning in different ways, as technical area learners have the tendency to use more mobile technology for studying purposes (Trivedi, 2024).

Furthermore, research studies socio-psychological impacts produced by social media on university students in Lahore, Pakistan. Although pointing out such negative aspects as the change of social behavior and health risk, the study also underscores the positive role social media performs in student social connection and educational resources (Zawar et al., 2023). The impact of phubbing on academic engagement (a phenomenon where people tend to use a smartphone instead of interacting with others) are also studied (Kobicheva et al., 2025). According to the studies, excessive smartphone use, particularly in the presence of phubbing, slopes cognitive engagement and academic success. In addition, it indicates that purposeful usage of smartphones may increase academic engagement, as the need for healthy usage habits is persistent. The balance can be defined by such aspects as students' level of time management skills and the requirements of the courses and so on, because various students manage mobile device usage better.

Al-Nuaimi et al. (2021) found out that excessive smartphone and social media use support procrastination and alienation from academic duties, finally having an impact on academic success and mental health. Further, they analyze aspects including personal dispositions of students such as impulsiveness and their socio-economic conditions, assuming that these are relevant to the experience and management of digital distractions. This study develops evidence of the negative effect of excessive use of digital devices on students' academic outcomes and psychological state. Additionally, they note that responsible use of ICT can enhance learning of students, by enabling better collaboration and availability of learning materials.

Kozhukhar and Belousova (2021) study internet addiction in Moscow schoolchildren and the impact of internet addiction on the socio-psychological adaptation of schoolchildren. The research finds out that more addicted students tend to experience increased emotional discomfort, maladjustment, and social/psychological inability to adjust. The study continues to examine the influence of status, socio-economic status more specifically, in that it makes students in less desirable economic backgrounds more vulnerable to negative impacts from internet addiction due to additional stressors. Besides, they note that proper use of the internet can make important educational materials easily accessible and support useful social interaction.

In addition, Bangura examines the impact of social communication on academic performance, especially in a sociological student body of the University of Sierra Leone. The study reports that although social communication and digital networking can promote cooperative learning and promote academic engagement, social communication and digital networking may distract focus if not well controlled. There is a wide range of influences of mobile phones and social media on academic performance depending on the students' academic discipline and age. This research confirms the assumption that the effect of social media and digital communication on students' academic success and socio-psychological wellbeing depends on the way in which they are used, but the potential is both positive and negative (Bangura, 2024).

The studies of students' use smartphones and the internet demonstrate both the positive and negative impact of their use. As much research points out harmful effects such as addiction, less school engagement, and socio-psychological problems, an increasing awareness of the potential benefits is developing. Cell phones and digital platforms can be used to impart education, interaction and development, if these are utilized properly. Adopting moderation in the consumption of technology is the central lesson learnt from literature, to enhance its good and reduce its possible bad. In addition, it is necessary to examine what impact that dependent factors like age, social and economic background, field of study, and ability to manage time have on the outcomes of smartphone and internet usage for students. There is reason why current research should continue to observe how digital technology continues to evolve so fast and, become an important part of education in that digital technology should be utilized safely and effectively to improve academic performance and wellbeing.

### Significance of the study

This study is significant as it provides critical insights for students, teachers, parents, and government officials regarding the various dimensions of mobile phone usage and its impacts, whether beneficial or harmful. Understanding the misuse of mobile phones will help discourage negative usage patterns. The primary importance of this research is to highlight the appropriate use of mobile phones and to prevent their misuse and overuse, which can be detrimental to all students, particularly university students. The economic, social, psychological, and sometimes moral impacts of excessive mobile phone use are significant, influencing overall academic performance. This study aims to bring about revolutionary changes by serving as a foundation for future research in this field. It will guide students toward the proper and positive use of mobile phones within an educational setup, ultimately fostering greater interest in studies and enhancing academic performance. The implications of mobile phone use among university students are crucial for several reasons. Firstly, mobile phones have fundamentally altered the dynamics of interpersonal communication, facilitating instant connectivity across distances but potentially diminishing the quality of face-to-face interactions. Secondly, the psychological effects of mobile phone use, including addiction, anxiety, and sleep disturbances, are increasingly recognized as significant concerns among young adults. Lastly, in academic settings, mobile phones pose challenges related to distraction, academic integrity, and their impact on learning and performance.

### Objective of the study

1. To analyze the consequences of excessive mobile phone usage on the learning and academic performance of students.
2. To identify the various socio-psychological impacts of mobile phones on students.

### Conceptual framework

The conceptual framework is structured to scrutinize the relationships between different variables (Table 1).

**Table 1:** The framework designed to investigate the relationships between different variables.

**Independent variables**	**Dependent variable**
• Lacking interest in studies. • Less participation in class and extra curriculum activities. • Mentally absent in the lecture room due mental health. • Causes of psychological & health related issues.	Students' academic performance

### Theoretical framework

#### The constructivist theory of mobile phone technology in education

The Constructivist Theory in education asserts that learners actively construct their own understanding and knowledge of the world through experiences and reflection on those experiences. When applied to mobile phone technology in education, this theory suggests that mobile phones can be powerful tools for learning, as they enable students to interact with content in a hands-on and personalized manner. Here's how Constructivist Theory specifically applies mobile phone technology in education:

##### Active learning

Mobile phones facilitate active learning by allowing students to engage directly with educational content. They can access information, participate in interactive learning activities, and collaborate with peers in real-time. The process can be facilitated, or it can be hindered, and it depends on such variables as the technological background of the student, and the focus of their studies (STEM vs. Humanities, for example).

##### Personalized learning

Mobile technology enables personalized learning by adapting content to fit each student's individual learning needs and preferences. Applications and platforms can provide content that matches the student's level of understanding and learning pace. However, the extent to which such personalization takes place may be influenced by aspects such as access to educational technology that also varies with socio-economic background.

##### Collaborative learning

Mobile phones support collaborative learning experiences, enabling students to work together on projects, share resources, and communicate regardless of their physical location. This promotes social interaction and collective problem-solving.

##### Contextual learning

Mobile phones facilitate contextual learning by connecting theoretical knowledge to real-world situations. Students can use apps and tools that simulate or relate to actual scenarios relevant to their studies.

##### Reflection and feedback

Mobile technology supports reflection on learning experiences by providing tools for documenting progress, receiving feedback, and revising understanding over time. This helps in refining knowledge construction.

##### Multimedia integration

Mobile phones enable the integration of various multimedia elements such as videos, audio recordings, images, and interactive simulations. This rich media environment enhances engagement and supports multiple learning modalities.

##### Access to resources

Mobile phones provide easy access to a vast array of educational resources, including e-books, online courses, academic journals, and educational apps. This supports independent learning and exploration.

##### Continuous learning

Mobile phones allow learning to extend beyond the classroom and traditional school hours. Students can continue their education asynchronously, accessing materials and resources at any time that suits their learning schedules.

In essence, Constructivist Theory supports the view that learning is an active and evolving process, and mobile phone technology can be leveraged to empower students in constructing their own knowledge. By engaging students in meaningful activities that involve exploration, interaction, collaboration, and reflection, mobile phones can transform educational experiences and enhance learning outcomes (Sung et al., 2016). According to the theory, Socioeconomic background, age, and academic discipline are one of the key determinants of mobile phone utilization for educational purposes that should be considered.

## Materials and methods

This research was conducted at two public sector universities: Guizhou Normal University and Guizhou University. These institutions were selected to analyze the implications of mobile phone usage on the socio-psychological and academic performance of university students. A quantitative research approach was employed, utilizing a cross-sectional survey design.

A two-stage probability sampling technique was used for participant selection. The process involved:

1. **Stratified sampling:** The student population was divided into strata based on relevant characteristics.
2. **Simple random sampling:** Within each stratum, students were randomly selected to ensure representativeness.

A total of 300 students were selected for the study, with 150 students from each university. This equal representation allows for a balanced comparison of mobile phone use and its socio-psychological and academic effects across both institutions. The survey was constructed so that variables such as gender, socioeconomic status and course discipline (STEM or humanities) could be controlled for while allowing for a careful analysis of the relationship between mobile phone use and academic and socio-psychological outcomes.

## Results and discussion

### Distribution of respondents by institution

The survey is distributed between participants Guizhou Normal University and Guizhou University. The sample comprised of 300 students from which Guizhou Normal University and Guizhou University received the same number of respondents at 150 representatives each. The equal representation in the samples makes it easy to make a systematic comparison of the impact of a mobile phone on socio-psychological and academic performance of students in the two universities.

Although, it is possible that socioeconomic status, and access to educational resources at each institution might moderate mobile phone use among students. Analysis of these factors, particularly how they function as confounding measures, will shed more light on the relation between the use of mobile phones and academic performance.

#### Analysis

The educational backgrounds of survey participants are provided in Table 2. Students' education is subdivided into four categories: high school, bachelors, masters, and doctoral students. Of the 300 participants, most of them, 99 students (33.0%), came from the high school section. This large sample size gives critical views related to the impact of mobile phone use on younger students at a critical stage of academic growth.

**Table 2:** Statistical analysis of educational level of students.

**Educational level**	**Frequency**	**%**	**Cumulative %**
High school section students	99	33.0	33.0
Bachelor's degree students	92	30.7	63.7
Master degree students	75	25.0	88.7
Doctor degree students	34	11.3	100
Total	300	100.0	

Influential factors of academic progress in students through mobile phone use can be diverse academic stages. For example, high-school students' experience can be more disrupted and academic performance—worse with using mobile phones compared to graduate students who usually have more defined academic priorities. Also, there might be a difference in the way mobile phones are used which affects the emotional and wellbeing of students between the educational levels—students at high school may be more affected by peer influence and graduate students more.

The largest group of respondents consists of high school students, making up 33.0% of the sample. This significant representation allows for a comprehensive understanding of how mobile phone usage impacts younger students who are at a critical stage of their academic journey. The high percentage indicates that mobile phone usage patterns and their effects are relevant concerning the high school level. Bachelor's degree students represent 30.7% of the respondents. This group is essential for analyzing the impact of mobile phone usage on undergraduate students, who are often balancing academic responsibilities with increased social interactions facilitated by mobile phones. Understanding the effects at this level can help develop targeted interventions to enhance academic performance and wellbeing. Master's degree students comprise 25.0% of the respondents. Graduate students typically have more specialized academic goals and greater independence in their studies. Analyzing this group's mobile phone usage provides insights into how advanced students manage their academic work and the potential benefits or drawbacks of mobile technology in their studies.

### Time consumption of mobile phone use

#### Analysis

Table 3 indicates how respondents spread out about daily use of mobile phone. The analysis implies that usage patterns vary among the sampled 300 participants.

- About a third of all respondents reported that during the daytime they use the mobile phone for 1–2 h.
- An impressive 31.7% of the respondents indicated that they are using their mobile phones for 3–4 h of a day.
- Every day, 26.7% of the respondents use their mobile phone for 5–6 h.
- Another 12.7% reported spending more than 6 h using their mobile phone each day.

**Table 3:** Time consumption of mobile phone use.

**Time consumption**	**Frequency**	**%**	**Cumulative %**
1–2 h	87	29.0	29.0
3–4 h	95	31.7	60.7
5–6 h	80	26.7	87.3
More	38	12.7	100.0
Total	300	100.0	

Such issues as age, gender, and the domain of subject's students are studying (STEM vs. humanities) can be the reasons for the differences in the time spent using mobile phones. For example, it is typical that students of the STEM disciplines frequently reference their mobile phones in an educational context, while students of the humanities are more likely to be absorbed by social media. In addition, socioeconomic status may contribute to the usage of mobile phones among the students in the high class may use their phones more since they can access advanced technology paralleling findings in recent literature (Kozhukhar and Belousova, 2021).

The rise in the use of mobile devices, which can now take over 5–6 h a day, is likely to create a significant influence on the academic as well as on the mental health sphere. According to the data, it might lead to concentration issues, increased procrastination, and increased level of stress. Consequently, analyzing these moderating variables plays a crucial role in the assessment of the degree to which the use of mobile phones affects students.

Table 4 depicts five different items of statement through which data were analyzed. This item includes mobile phones having negative impacts on students' moral aspects, mental absence in lectures, mobile use making them less participation in class, extra and unwanted use of mobile, sometimes failing papers, and students spending mobile phones rather than taking books (Al-Nuaimi et al., 2021). The responses of the students with the use of mobile phones in a ratio of 126 (42.0%), 66 (22.0%) agreed (A) and strongly agreed (SA), and 53 (17.7%) were neutral (N) in their responses in the statement that mobile phones have put negative impacts on student's moral aspects. Likewise, 122 (40.7%) and 62 (20.7%) agreed (A) and strongly agreed (SA), while 65 (21.7%) were in neutral (N) categories in the statement of mobile phone users mentally absent in lectures due to late-night packages. Correspondingly, 140 (46.7%), 58 (19.3%) agreed (A) and strongly agreed (SA), respectively, while 47 (15.7%) respondents were in neutral (N) categories in the table statement of excessive users of mobile phones by the students make them less participation in class. The table further explores the evidence of respondents in a ratio of 136 (45.3%), 59 (19.7%) agree (A) and strongly agree (SA) in the statement of extra and unwanted usage of mobile by the students sometimes failed in the paper. Evidence from the table shows that 110 (36.7%), 95 (31.7%) agree (A) and strongly agree (SA), respectively. In comparison, 49 (16.3%) were neutral (N) in a statement that students often spent money on mobile expenses rather than taking books and academic materials.

**Table 4:** Response category negative factors.

**Response item**	**SD**	***D***	***N***	***A***	**SA**	**Total**
Mobile has negative impacts on students' moral aspects	17 (5.7)	38 (12.7)	53 (17.7)	126 (42.0)	66 (22.0)	300
Mobile phone users mentally absent in lecture due to late night packages	17 (5.7)	34 (11.3)	65 (21.7)	122 (40.7)	62 (20.7)	300
Excessive use of mobile by the students make them less participation in class	13 (4.3)	42 (14.3)	47 (15.7)	140 (46.7)	58 (19.3)	300
Extra and unwanted usage of mobile by the students sometimes failed in paper	18 (6.0)	39 (13.0)	48 (16.0)	136 (45.3)	59 (19.7)	300
Students often spend money on mobile expenses rather than taking books	16 (5.3)	30 (10.0)	49 (16.3)	110 (36.7)	95 (31.7)	300

Statistical analysis of response categories related to negative factors was carried out through five different statement items shown in Table 5. Most participants agreed that mobile phones are being used in the examination hall as tools for unfair practices. (i) The students' priority is to make calls and SMS replies, instead of doing some homework. For most students, working will be focused on making calls and SMS responses left before completing their homework. (ii) Mobile phone users are mostly not actively involved in extra campus activities. The fourth statement, related to the effects of WeChat and SMS on language skills, indicated that 127 (42.7%) agreed and 68 (23.0%) strongly agreed, whereas 166 (55.7%) agreed with the fifth statement on mobile phone misuse.

**Table 5:** Response category negative factors.

**Response item**	**SD**	***D***	***N***	***A***	**SA**	**Total**
Students use mobile in the examination hall as source of unfair means	15 (5.0)	40 (13.3)	46 (15.3)	106 (35.0)	93 (31.0)	300
Students' priority is to make call and SMS reply than to do homework	13 (4.3)	38 (12.7)	43 (14.3)	141 (47)	65 (21.7)	300
Mobile phone usage students did not participate in extracurricular activities	15 (5.0)	33 (11.0)	70 (23.3)	131 (43.0)	51 (17.0)	300
Use of WeChat and SMS deteriorates students' language capabilities	17 (5.7)	32 (10.7)	40 (13.3)	125 (41.0)	86 (28.7)	300
Most of the students misuse mobile phone	19 (6.3)	27 (9.3)	49 (16.3)	129 (43.0)	76 (25.3)	300

As far as the first statement is concerned, students use mobile phones in the examination hall as a source of unfairness, 106 (35.3%) participants agreed, 93 (31.0%) strongly agreed, and 46 (15.3%) were neutral. Students give priority attention to making phone calls and receiving SMS responses over homework according to the second statement with 141 (47.0%) in agreement and 65 (21.7%) in strong agreement. Most of the respondents reported that the student avoids extracurricular activities. Responding, 131 (43.7%) agreed, an additional 51 (17.0%) strongly agreed with the results.

Participants agreed that using WeChat and SMS too often weakens students' language skills. Out of 125 (41.7%), 86 (28.7%) strongly agreed, 40 (13.3%) neither agreed nor disagreed. According to its fifth point, most students tend to abuse their mobile phones. One hundred and twenty-nine (43.0%) of the participants agreed, 76 (25.3%) agreed strongly and 49 (16.3%) were neutral to this statement (Table 6).

**Table 6:** Response category socio-psychological factors.

**Response item**	**SD**	***D***	***N***	***A***	**SA**	**Total**
Mobile use by students all the time leads to psychological illness	18 (6.0)	30 (10.0)	57 (19.0)	134 (44.7)	61 (20.3)	300
Excessive involvement with mobile creates relationship crises	10 (3.3)	28 (8.3)	46 (15.3)	142 (47.73)	77 (25.3)	300
Mobile phone uses by the students degraded their moral values	16 (5.3)	49 (16.3)	57 (19.0)	199 (39.7)	59 (19.7)	300
Excess use of mobile isolate students from their fellows	10 (3.3)	20 (6.7)	46 (15.3)	141 (47.0)	83 (27.7)	300
Students rely heavily on the internet create problems of emotional exhaustion	14 (4.7)	26 (8.7)	48 (16.0)	132 (44.0)	80 (26.7)	300

Table 6 indicates the differences in responses in statistical values which was related to Nwanzu and Babalola (2023). Data was investigated under five socio-psychological response categories such as constant mobile use by students causing psychological illness, high mobile usage causing conflict in peer relationships, decline in morality in students, constant mobile use alienating students from their peers, and excessive use of the internet causing emotional burnout. Among respondents, 134 (44.7%) agreed and 61 (20% disagreed. Most of the students, 142 students (47.73%), agreed that too much mobile phone usage could initiate relationship issues with fellows. A majority out of 199 participants (39.7%) said that mobile phones use has a negative influence on their moral values. Many participants 141 (47.0%) and 83 (27.7%) resounded that excessive use of mobile phones creates isolation among school students. Many of them, 80 (26.7%) of strong agreement and 132 (44.0%) of agreement, believe that students' internet dependency is a major cause of emotional exhaustion. The results match both the explanations from Selye and the distraction ideas from Klingsieck (Sung et al., 2016; Klingsieck, 2021).

Table 7 lists the statistical information concerning time management students' time-wasting on unnecessary texting or messaging friends or colleagues using phones, and it takes time that could be spent on studying, impact of use of WeChat on academic performance of students, watching the films or music beyond the classroom hours, and the negative impact. The opinions of the respondents were investigated and most of 136 (45.3%) and 88 (29.3%) agreed and strongly agreed on the basis that the use of mobile phones leads to a waste of time in writing or sending unproductive SMS to friends or colleagues.

**Table 7:** Response category time factors.

**Response item**	**SD**	**D**	**N**	**A**	**SA**	**Total**
Students waste time writing/sending useless messages to friends/colleagues	16 (5.3)	29 (9.7)	31 (10.3)	136 (45.3)	88 (29.3)	300
Mobile usage by the students leads toward wastage their study time	13 (4.3)	25 (8.3)	34 (11.3)	149 (49.7)	79 (26.3)	300
Mobile usage by the students WeChat leads toward the lower GPA	11 (3.7)	24 (8.0)	40 (13.3)	124 (41.3)	101 (33.7)	300
Mobile uses by students watching movies and songs outside the class	11 (3.7)	27 (9.0)	36 (12.0)	135 (45.0)	91 (30.3)	300
Students use mobiles in excess leads toward the lack of sleeping	11 (3.7)	27 (10)	38 (14.7)	134 (48.7)	90 (23.0)	300

In addition, from the table it is possible to find out that the majority of 149 (49.7%) and 79 (26.3%) agreed or strongly agreed that students spend more time on mobile phones than studying.

Most students indicated agreement or strong agreement with the answer (124 or 41.3% and 101 or 33.7%), whereas 40 students or 13.3% remained neutral toward the effect of WeChat on GPA.

Besides, 135 (45.0%) and 91 (30.0%) students agreed and strongly agreed they use mobile phones to watch movies or listen to songs when they are not in class. The majority of 134 respondents (48.7%) or 90 respondents (23.0%) reported agreement or strong agreement that excessive use of mobile phones by students leads to problems such as sleep deprivation. Mobile phone overuse and poor sleep are linked by Nakshine et al. (2022), who used meta-analytic methods to find a relationship between extended screen time and reduced sleep health and mental health.

Chi-square and Gamma tests on various factors are reported in Table 8. The correlation between certain psychological factors and coordination factors is statistically significant (*X*^2^ = 147.918), as confirmed by a very low *p*-value (*P* = 0.004) and a very weak, negative Gamma value (−0.08). The research also found that psychological aspects are closely connected to positive aspects (*X*^2^ = 297.742), in a way that is significant (*P* = 0.002) and weakly positive (Gamma = 0.05). This means there is a small link between better phone use by students and reduced mental issues. Likewise, greater psychological and negative aspects are related (*X*^2^ = 450.450, *P* = 0.015), with a Gamma (−0.35) showing that the more positive effects mobile phones have, the more psychological difficulties and poor academic results emerge (Klingsieck, 2021). The study also found a connection between time and psychological aspects (*X*^2^ = 245.487, *P* = 0.007), with a correlation of −0.09, meaning that investing additional time on mobile devices might negatively influence someone's psychological state.

**Table 8:** Association between psychological aspects and academic factors.

**Attributes**	**Factors**	***X* ^2^**	**Sig. value (*p*)**	**Gamma value**
Psychological aspects	Co-ordination aspects	147.918	0.004	−0.08
	Positive aspects	297.742	0.002	0.05
	Negative aspects	450.450	0.015	−0.35
	Time aspects	245.487	0.007	−0.09

The statistics suggest that anxiety and depression can cause serious problems with a student's grades and performance related to recent studies (Neophytou et al., 2021). When anxiety is prolonged, students find it more difficult to focus and recall and when depression is prolonged, their motivation and energy aren't strong enough to help them succeed academically.

Table 9 reveals the impact and direction of the relationship between mobile phone use and related factors. Based on the study outcomes, there is a significant statistical relationship between time aspects and coordination aspects (*p*-value of 0.004), weakly pointed in the negative direction (Gamma = −0.07). A review of time and positive aspects also points to a connection with a chi-square test statistic of 355.45 and a *p*-value of 0.003 which is significant. There is a strong relationship between time and negative factors (*X*^2^ = 386.17, *P* = 0.015) and this relationship is moderately negative (Gamma = −0.30). Such is the case with time and psychological aspects as well (*X*^2^ = 429.60, *P* = 0.001), with Gamma showing only a slight negative connection (−0.08). Evidence shows that using mobile phones for a long time each day reduces your ability to manage your time well and affects your school grades.

**Table 9:** Association between time aspects and academic factors.

**Attributes**	**Factors**	***X* ^2^**	**Sig. value (*p*)**	**Gamma value**
Time aspects	Co-ordination aspects	142.89	0.004	−0.07
	Positive aspects	355.45	0.003	0.06
	Negative aspects	386.17	0.015	−0.30
	Psychological aspects	429.60	0.001	−0.08

Table 10 indicates the relationship between how much time people spend using their mobile phones and their beliefs about coordinating their academic tasks. All the information for each category was coded and put onto the table. It appears that participants' use of mobile phones affects how they coordinate with one another. A below-critical value of *P* = 0.031 was shown in the relationship between these two variables (*X*^2^ = 18.77). The relationship between How often people play mobiles and coordination is small and suggests that there is little impact on coordination from people spending time on their phones.

**Table 10:** Association between time consumption and academic factors.

**Attributes**	**Factors**	***X* ^2^**	**Sig. value (*p*)**	**Gamma value**
Time consumption (1–2 h, 3–4 h, 5–6 h)	Co-ordination aspects	18.77	0.031	−0.04
	Positive aspects	19.41	0.028	0.07
	Negative aspects	14.25	0.043	−0.02
	Psychological aspects	14.55	0.039	0.05
	Time aspects	15.72	0.035	−0.09

Time spent is linked to positive academic benefits with only a weak connection, but this effect is statistically significant (*X*^2^ = 19.41, *P* = 0.028), as shown by the connecting value of 0.07. It appears that mild use of mobile phones might help students improve in certain aspects of learning.

Having more time on mobile phones shows only a weak or negligible connection to having negative academic results (*X*^2^ = 14.25, *P* = 0.043). Additionally, time spent on mobile phones correlates with psychological factors in a statistically meaningful way, with a weak but positive Gamma value. Furthermore, how much time is spent on the phone is significantly connected to time aspects (*X*^2^ = 15.72, *P* = 0.035), as the Gamma value is small and negative (−0.09), so longer use of phones may sometimes negatively affect both time management and grades.

According to these results, the negative effects of increased mobile phone use on studying and mental health are unlikely to be strong. The mixed results in the research, relating valuable learning from phones to lower academic scores, agree with Ibrahim and Jibia's (2024) connectivism theory which supports the impact of tech on learning, but also brings distractions. However, excessive use may contribute to emotional exhaustion, relationship issues, sleep disturbances, and procrastination, which can negatively impact academic achievement and increase stress levels. Researchers recommend interventions focused on improving time management and reducing procrastination to enhance academic success and mental health.

## Conclusion

The study was formulated to determine the effect of mobile phone habits among students on their academic performance, social behaviors, mental health, paying a special attention to the university students in Guizhou province, China. The main question involved investigating how students use mobile phones, irrespective of purpose, influenced their academic success and socio-psychological health. Results from the study highlight that excessive use of mobile devices for social media, games and chatting is associated with bigger psychological challenges and worsened coordination in studying and poorer academic performance for students. It is reported that overuse of phones predicts lower involvement in academic pursuit, lower test scores, higher anxiety, and sleep problems. Using mobile phones for educational purposes such as educational materials access or group assignment completion by students resulted in positive effects with a slight positive correlation to better psychological wellbeing and academic engagement, emphasizing the importance of productive use. The research indicates that how much time people spend with their phones tends to weaken academic focus and general wellbeing, though not importantly. The present study also focuses on the impact of moderating factors such as age, gender, academic discipline distinction (e.g., stem/humanities), and socioeconomic context. Identification of the role of these factors in students' mobile technology interactions allows developing individualized intervention that promotes healthy usage patterns and reduces possible negative effects. This investigation advances the body of knowledge about the social and psychological effects and academic implications of mobile phone use in students. It provides effective data for educators, policymakers and mental health field workers committed to fostering positive behavior as it relates to mobile phones in educational environments. Exploring the issue of moderation and awareness, this research presents an invitation for more research into strategies to use technology to enhance learning without compromising the emotional health of students.

## Data Availability

The original contributions presented in the study are included in the article/supplementary material, further inquiries can be directed to the corresponding authors.

## References

[B1] Al-Nuaimi M. N. Bouazza A. Abu-Hilal M. M. (2021). Parameters of ICT-associated deviant behaviour among Omani undergraduates: a socio-psychological perspective. Global Knowl. Memory Commun. 70, 225–253. 10.1108/GKMC-12-2019-0148

[B2] Bangura M. (2024). Sociological upshot of social communications on the academic performance of sociology students at University of Sierra Leone, Fourah Bay College. Eur. J. Contemp. Educ. E-Learn. 2, 3–17. 10.59324/ejceel.2024.2(1).01

[B3] Campbell S. W. Park Y. J. (2008). Social implications of mobile telephony: the rise of personal communication society. Sociol. Comp. 2, 371–387. 10.1111/j.1751-9020.2007.00080.x

[B4] Chen Z. Chen W. Jia J. An H. (2020). The effects of using mobile devices on language learning: a meta-analysis. Educ. Technol. Res. Dev. 68, 1769–1789. 10.1007/s11423-020-09801-5

[B5] Duncan D. K. Hoekstra A. R. Wilcox B. R. (2012). Digital devices, distraction, and student performance: does in-class cell phone use reduce learning. Astron. Educ. Rev. 11, 1–4. 10.3847/AER2012011

[B6] Gündüz U. (2017). The effect of social media on identity construction. Mediterran. J. Soc. Sci. 8:26. 10.1515/mjss-2017-0026

[B7] Hartley K. Hoffman B. Andújar A. (2023). Smartphones and learning: evaluating the focus of recent research. Eur. J. Invest. Health Psychol. Educ. 13, 748–758. 10.3390/ejihpe13040056 37185909 PMC10138152

[B8] Hashemi S. Ghazanfari F. Ebrahimzadeh F. Ghavi S. Badrizadeh A. (2022). Investigate the relationship between cell-phone over-use scale with depression, anxiety and stress among university students. BMC Psychiatry. 22:755. 36460976 10.1186/s12888-022-04419-8 PMC9716159

[B9] Ibrahim T. A. Jibia S. A. (2024). Utilizing Technology for Education: Exploring Innovative Ways in Education To Bridge The Digital Divide In Underserved Communities. International Journal of Library Science and Educational Research. Cambridge Research and Publications.

[B10] Junco R. Cotten S. R. (2012). No A 4 U: the relationship between multitasking and academic performance. Comput. Educ. 59, 505–514. 10.1016/j.compedu.2011.12.02320235414

[B11] Klingsieck K. B. (2021). Procrastination in different life-domains: is procrastination domain specific? Curr. Psychol. 40, 1270–1287.

[B12] Kobicheva A. Tokareva E. Baranova T. (2025). How does students' level of phubbing relate to academic engagement and performance variables? Eur. J. Psychol. Educ. 40, 1–16. 10.1007/s10212-024-00905-7

[B13] Kozhukhar G. Belousova A. (2021). Level of internet addiction of schoolchildren and specific character of their socio-psychological adaptation. E3S Web of Conf. 258:07069. 10.1051/e3sconf/202125807069

[B14] Kuznekoff J. H. Titsworth S. (2013). The impact of mobile phone usage on student learning. Commun. Educ. 62, 233–252. 10.1080/03634523.2013.767917

[B15] Madigan S. Eirich R. Pador P. McArthur B. A. Neville R. D. (2022). Assessment of changes in child and adolescent screen time during the COVID-19 pandemic: a systematic review and meta-analysis. JAMA Pediatrics 176, 1188–1198. 10.1001/jamapediatrics.2022.4116 36342702 PMC9641597

[B16] Marinucci M. Pancani L. Aureli N. Riva P. (2022). Online social connections as surrogates of face-to-face interactions: a longitudinal study under Covid-19 isolation. Comput. Hum. Behav. 128:107102. 10.1016/j.chb.2021.107102

[B17] Moe D. J. (2024). Effects of Non-Educational Technology on Adolescents' Well-Being and School Performance. Bethel University, Saint Paul, MN.

[B18] Nakshine V. S. Thute P. Khatib M. N. Sarkar B. (2022). Increased screen time as a cause of declining physical, psychological health, and sleep patterns: a literary review. Cureus 14:e30051. 10.7759/cureus.30051 36381869 PMC9638701

[B19] Neophytou E. Manwell L. A. Eikelboom R. (2021). Effects of excessive screen time on neurodevelopment, learning, memory, mental health, and neurodegeneration: a scoping review. Int. J. Mental Health Addict. 19, 724–744. 10.1007/s11469-019-00182-2

[B20] Nwanzu C. L. Babalola S. S. (2023). Socio-psychological factors in sustainable mobile phone facilities usage behaviour. Int. J. Mobile Commun. 21, 410–431. 10.1504/IJMC.2023.13001335009967

[B21] Sung Y. T. Chang K. E. Liu T. C. (2016). The effects of integrating mobile devices with teaching and learning on students' learning performance: A meta-analysis and research synthesis. Comput. Educ. 94, 252–275.

[B22] Thomée S. Härenstam A. Hagberg M. (2011). Mobile phone use and stress sleep disturbances, and symptoms of depression among young adults: a prospective cohort study. BMC Public Health 11:66. 10.1186/1471-2458-11-66 21281471 PMC3042390

[B23] Trivedi B. V. (2024). An examination of cellphone usage and its impact on students: a social work approach. Vidhyayana-An Int. Multidiscipl. Peer-Rev. E-J. 9.

[B24] Walters A. A. Hunsicker-Walburn M. J. (2015). Exploring perceptions of technology's impact on academic misconduct. J. Appl. Res. Higher Educ. 7, 32–42. 10.1108/JARHE-02-2014-0024

[B25] Wong C. W. Tsai A. Jonas J. B. Ohno-Matsui K. Chen J. Ang M.. (2021). Digital screen time during the COVID-19 pandemic: risk for a further myopia boom? Am. J. Ophthalmol. 223, 333–337. 10.1016/j.ajo.2020.07.034 32738229 PMC7390728

[B26] Yildirim C. Correia A. P. (2015). Exploring the dimensions of nomophobia: development and validation of a self-reported questionnaire. Comput. Hum. Behav. 49, 130–137. 10.1016/j.chb.2015.02.059

[B27] Zawar S. Malik A. S. Sultan H. (2023). Beyond likes and shares: analyzing social media's impact on socio-psychological behavior of university students of Lahore, Pakistan. Online Media Soc. 4, 1–11. 10.71016/oms/0yken568

